# Selenomethionine Suppressed TLR4/NF-κB Pathway by Activating Selenoprotein S to Alleviate ESBL *Escherichia coli*-Induced Inflammation in Bovine Mammary Epithelial Cells and Macrophages

**DOI:** 10.3389/fmicb.2020.01461

**Published:** 2020-07-08

**Authors:** Cuicui Zhuang, Gang Liu, Herman W. Barkema, Man Zhou, Siyu Xu, Sadeeq ur Rahman, Yongxia Liu, John P. Kastelic, Jian Gao, Bo Han

**Affiliations:** ^1^Department of Clinical Veterinary Medicine, College of Veterinary Medicine, China Agricultural University, Beijing, China; ^2^Department of Production Animal Health, Faculty of Veterinary Medicine, University of Calgary, Calgary, AB, Canada; ^3^Section of Microbiology, Department of Pathobiology, College of Veterinary Sciences and Animal Husbandry, Abdul Wali Khan University, Mardan, Pakistan; ^4^College of Veterinary Medicine, Shandong Agricultural University, Tai’an, China

**Keywords:** selenomethionine, ESBL-*Escherichia coli*, bovine mastitis, inflammation, selenoprotein S, TLR4/NF-κB pathway

## Abstract

Inflammation is the hallmark of extended-spectrum β-lactamase (ESBL)-producing *Escherichia coli*-induced bovine mastitis. Organic selenium can activate pivotal proteins in immune responses and regulate the immune system. The present study aimed to investigate whether selenomethionine (SeMet) attenuates ESBL *E. coli*-induced inflammation in bovine mammary epithelial cells (bMECs) and macrophages. Cells were treated with 0, 5/10, 10/20, 20/40, or 40/60 μM SeMet for 12 h and/or inoculated with ESBL*-E. coli* [multiplicity of infection (MOI) = 5] for 4/6 h, respectively. We assessed inflammatory responses, including selenoprotein S (SeS), Toll-like receptor 4 (TLR4), Ikappa-B (IκB), phospho-NF-κB p65 (Ser536), interleukin-1β (IL-1β), tumor necrosis factor-α (TNF-α), and lactate dehydrogenase (LDH) activities. Treatment with 40/60 μM SeMet promoted cell viability and inhibited LDH activities in both bMECs and macrophages. Inoculation with ESBL-*E. coli* reduced cell viability, which was attenuated by SeMet treatment in bMECs and macrophages. SeMet increased ESBL *E. coli*-induced downregulation of SeS and decreased LDH activities, TLR4, IκB, phospho-NF-κB p65 (Ser536), IL-1β, and TNF-α protein expressions in bMECs and macrophages. In addition, knockdown of SeS promoted protein expression of TLR4-mediated nuclear factor-kappa (NF-κB) pathway and BAY 11-708 inhibited TNF-α and IL-1β protein levels in bMECs and macrophages after ESBL-*E. coli* treatment. Moreover, ESBL-*E. coli* inoculation increased monocyte chemoattractant protein 1 (MCP-1), C–C motif ligand 3 (CCL-3), and CCL-5 mRNA expressions in bMECs. In conclusion, ESBL-*E. coli* induced expression of MCP-1, CCL-3, and CCL-5 in bMECs and then recruited and activated macrophages, whereas SeMet attenuated ESBL *E. coli*-induced inflammation through activated SeS-mediated TLR4/NF-κB signaling pathway in bMECs and macrophages.

## Introduction

*Escherichia coli* is an opportunistic environmental pathogen affecting bovine mammary glands, causing clinical mastitis, particularly during early lactation in dairy cows (Olde Riekerink et al., 2008; Gao et al., 2017). Prevalence of highly resistant extended-spectrum β-lactamase (ESBL)-producing *E. coli* is increasing in food-producing animals in various countries, including China (Liu et al., 2014; Ali et al., 2016; Zhuang et al., under review). The high incidence of multidrug-resistant *E. coli* strains is problematic, as treatment options are limited and severe complications are common (Mukherjee et al., 2013). Therefore, a better understanding of the pathogenesis of this major bacterial disease is needed.

*Escherichia coli* penetrates the lumen of the mammary gland, promoted by lactate dehydrogenase (LDH) release (Vangroenweghe et al., 2005) and incites distinct inflammatory and innate immune responses (Gilbert et al., 2013). When infection occurs, mammary epithelial cells and entry of macrophages into the mammary gland are initial responses against mastitis pathogens (Bougarn et al., 2011; Vural and Kehrl, 2014). Therefore, bovine mammary epithelial cells (bMECs), and macrophage lines are an ideal model to evaluate *in vitro* inflammation induced by *E. coli*. Furthermore, they are well characterized, readily available, and moderately priced. In addition, mammary epithelial cells and macrophages each have an array of germline-encoded pattern recognition receptors/sensors that recognize pathogen-associated molecular patterns and which activate the nuclear factor-kappa B (NF-κB) signaling pathway to mediate inflammatory and innate immune responses. In response to cell stimulation, NF-κB, which binds with Ikappa-B (IκB) protein in the cytoplasm, is released and translocates into the nucleus. Furthermore, the P65 subunit of NF-κB contains the transcriptional activation domain, which regulates transcription of inflammatory proteins during infection, e.g., tumor necrosis factor-α (TNF-α), and interleukin-1β (IL-1β; Lecoq et al., 2017).

Selenium (Se) is an essential trace element, with a Se-enriched diet being the major source of Se intake for mammals. To correct Se deficiency, recommended Se supplementation is 0.3, 0.15 to 0.2, and 0.1 mg/kg dry matter Se in the United States, Germany, and Britain, respectively (Salman et al., 2009). The major representative chemical forms of Se are inorganic Se (selenite and selenate) and organic Se [selenocysteine, selenomethionine (SeMet), methylselenocysteine, and Se-enriched yeast] (Gabel-Jensen and Gammelgaard, 2010). These Se forms are involved in various biochemical and physiological functions, including immunity, antioxidant function, and aging (Kubachka et al., 2017). In addition, Se can regulate the inflammatory process (Kaushal et al., 2014), and selenocompounds can influence macrophage functions (Shimohashi et al., 2000). Moreover, feeding Se-yeast increased percentage of *E. coli* killed by blood neutrophils (Cebra et al., 2003). However, protective effects and molecular mechanisms of SeMet on ESBL-*E. coli* infection in bMECs and macrophages remain unclear. Therefore, the present study established an *in vitro* experimental model of bMECs and macrophages exposed to ESBL-*E. coli* to determine whether SeMet conferred anti-inflammatory defense capacity via modulation of Toll-like receptor 4 (TLR4)/NF-κB signaling pathway.

## Materials and Methods

### Statement of Ethics

This study was conducted in accordance with ethical guidelines and standard biosecurity and institutional safety procedures of China Agricultural University (CAU; Beijing, China). Prior to the start of the study, ethical approval was granted by the Departmental Committee of the College of Veterinary Medicine, CAU.

### Reagents and Antibodies

SeMet, dimethyl sulfoxide (DMSO), 3-(4,5-dimethyl-thiazol-2-yl)-2,5-diphenyl tetrazolium bromide (MTT), and *N*-acetyl-l-cysteine were acquired from Sigma-Aldrich Chemical (cat # 1217470-45-5, 298-93-1 and 616-91-1, Sigma, St. Louis, MO, United States). LDH activity assay kit was purchased from Beyotime Biotechnology (Shanghai, China). Opti-MEM (cat #SH30265.01) and fetal bovine serum (FBS, cat #10099-141) were purchased from Gibco (Grand Island, NY, United States). Lipofectamine 2000 reagent was from Thermo Fisher Scientific (Waltham, MA, United States). TransScript First-Strand complementary DNA (cDNA) Synthesis SuperMix and SYBR Green PCR Core Reagents were purchased from TransGen Biotech (TransGen Biotech, Beijing, China). Trizol Reagent was bought from Invitrogen (Carlsbad, CA, United States). The siRNA duplexes for bovine and murine Selenoprotein S (SeS) and two siRNA negative controls were purchased from Sangon Biotech (Guangdong, China). Primary antibodies to TLR4 (cat #19811-1-AP, 1:1000), NF-κB (cat #10745-1-AP, 1:1000), SeS (cat #15591-1-AP, 1:500), IKB (cat #10268-1-AP, 1:1000), TNF-α (cat # 17590-1-AP, 1:1000), IL-1β (cat #16806-1-AP, 1:1000), GAPDH (cat # 10494-1-AP, 1:2000), and PCNA (cat # 10205-2-AP, 1:2000) were obtained from Proteintech (Proteintech, Wuhan, China), and primary antibodies to the phospho-NF-κB p65 (Ser536; p-p65; cat # bs-0982R, 1:1000) were obtained from Bioss (Beijing, China). Horseradish peroxidase (HRP)-conjugated goat anti-rabbit (1:5000) IgG antibodies was from CW Bio (Beijing, China).

### Bacterial Strains, Cell Culture, and Treatment

A strain of sequence type ESBL 410(4) *E. coli*, isolated from a milk sample of a dairy cow with clinical mastitis in Inner Mongolia (Ali et al., 2017), was used. This isolate induced the most profound changes in inflammation, oxidative stress, and apoptosis (Zhuang et al., under review). ST410(4) *E. coli* was re-established from frozen stocks by culturing on tryptose soya agar (Difco TM, Becton Dickinson, Sparks, MD, United States) supplemented with 5% defibrinated sheep blood and incubated at 37°C for 72 h, then sub-cultured on Lysogeny broth medium to mid-log phase. The bMECs line MAC-T (Shanghai Jingma Biological Technology, Shanghai, China) and murine macrophage cell line J774A.1 (Sigma-Aldrich, St. Louis, MO, United States) were cultured as previously described (Nakase et al., 2017; Shahid et al., 2017). The SeMet was solubilized in distilled water (concentration, 0.1 mol/L) for storage at −80°C and subsequently thawed and diluted with distilled water into working solutions of 200 and 400 μmol/L (μM). The bMECs and macrophages were cultured in Dulbecco’s modified eagle medium (DMEM) containing various concentrations of SeMet. After SeMet exposure for 12 h, bMECs and macrophages were infected with ESBL-producing ST410(4) *E. coli* at a MOI of 0 and 5 in 6-well plates for 4 and 6 h at 37°C, respectively. BAY 11-7082 (10/20 μM; Beyotime Biotechnology) was treated for 1 h before SeMet treatment. Furthermore, a blank control group with no *E. coli* or SeMet whatsoever was also included. Broth was centrifuged (10,000 × *g* for 10 min) and cells and supernatant were stored separately at −80°C until analyzed.

### Cell Viability Assay

Cell viability was determined using an MTT reduction assay. The bMECs and macrophages were incubated with 0, 5, 10, 20, 40, 60, 80, and 100 μmol/L SeMet for 12 h. DMEM solution containing 10% MTT was added to the treated cells in each well. Cells were incubated at 37°C for 4 h, supernatants were removed, and formazan crystals were dissolved in 150 μl of DMSO. Absorbance was recorded at a wavelength of 490 nm and reference wavelength of 630 nm on a 318 MC microplate reader (Shanghai Sanco Instrument, Shanghai, China). Each group was assayed 20 times (10 replicates per group and each sample assayed in duplicate).

### Determination of LDH Activity

The bMECs and macrophages were cultured in 96-well plates and incubated with 0, 5, 10, 20, 40, 60, 80, or 100 μmol/L SeMet for 12 h. Thereafter, treated cells were infected with one ESBL-producing *E. coli* [ST410(4)] for 4 or 6 h. After infection, spent medium bMECs and macrophages was collected and centrifuged (10,000 × *g* for 10 min) to detect LDH activities, using an LDH activity assay kit (Beyotime Biotechnology), according to the manufacturer’s instructions. Adsorption values were determined at 490 nm with a 318 MC microplate reader (Shanghai Sanco Instrument). Assay sensitivity was 9.0–5,000 U/L and coefficient of variation was 1.5%. Each group was assayed 20 times (10 replicates per group and each sample assayed in duplicate).

### MCP-1, C–C Motif Ligand 3 (CCL-3), and CCL-5 mRNA Expressions

Monocyte chemoattractant protein 1 (MCP-1), CCL-3, and CCL-5 mRNA expressions were analyzed by quantitative real-time reverse transcription-polymerase chain reaction (qRT-PCR). Total RNA was extracted from splenic lymphocytes using Trizol Reagent (Invitrogen) and total RNA was reverse-transcribed using TransScript First-Strand complementary DNA (cDNA) Synthesis SuperMix (TransGen Biotech), according to the manufacturer’s instructions. The PCR primers were designed according to the NCBI database (Table 1) and PCR was performed with a StepOnePlus Real-Time PCR System (Thermo Fisher Scientific) using SYBR Green PCR Core Reagents (TransGen Biotech). Amplification of β-actin mRNA was used as an endogenous control. There were 10 replicates per group and each sample was assayed in triplicate and a mean value was calculated. Data were analyzed according to the 2^–ΔΔCt^ method and results were expressed as relative mRNA levels. Each group was assayed 18 times (three replicates per group and each sample assayed in sextuplicate).

**TABLE 1:** Primer sequence and length of amplification target gene.

Gene	Number	Upstream and downstream primer sequence	Primer length (bp)	Product length (bp)
MCP-1	NM_174006.2	UP:5′-GTGCTCGCTCAGCCAGATGC-3′	20	119
		LOW:5′- GGACACTTGCTGCTGGTGACTC-3′	22	119
CCL-3	NM_174511.2	UP:5′-TGCTCTCGCCGTTCTCCTCTG-3′	21	93
		LOW:5′-GAGAAGCAGCAGGCCGTTGG-3′	20	93
CCL-5	NM_175827.2	UP:5′-CTGCCACTGCCTTCGCTGTC-3′	20	84
		LOW:5′-GCGTGGTGTCCGAGGCATATG-3′	21	84
β-Actin	NM_173979.3	UP:5′-CTTCCAGCCGTCCTTCCT-3′	18	105
		LOW:5′-TGTTGGCATACAGGTCCTTTC-3′	21	105

### Small Interfering RNA and Transfection of Cells

To knock down SeS, specific small interfering RNA (siRNA) duplexes targeting the bovine and murine SeS gene and two siRNA negative controls were purchased from Integrated DNA Technologies (Sangon Biotech). First, 2.5 nmol SeS siRNA and control siRNA were dissolved in 125 μl of DNase/RNase-free water and stored at −80°C. Then, SeS siRNA and control siRNA stock solution were diluted in Opti-MEM medium and then diluted siRNA was added to Lipofectamine 2000 reagent and incubated for 10 min in bMECs (1.25 μl of SeS siRNA and control siRNA, 30 μl of Opti-MEM medium, and 3 μl of Lipofectamine 2000 reagent) and macrophages (2.5 μl of SeS siRNA and control siRNA, 28.75 μl of Opti-MEM medium, and 3 μl of Lipofectamine 2000 reagent). Cells were treated with the siRNA directed against SeS and the scrambled control siRNA (final concentrations, 25 and 50 nM) using Lipofectamine 2000 reagent (Invitrogen) in Opti-MEM without serum, according to the manufacturer’s instructions, as previously described (Han et al., 2018). After 24 h, transfection medium was removed and 1 ml of DMEM with SeMet (20 and 40 μM) was added to recover cell growth for 12 h. Subsequently, cells were infected with ESBL-producing ST410(4) for 4 or 6 h and then cells were harvested and subjected to Western blotting.

### Western Blotting Analysis

The bMECs and macrophages were lysed in buffer containing 10 mM Tris–buffer (pH 7.5), 1 mM PMSF, 1% Triton X–100, 1 mM protease inhibitor cocktail, and 1 mM phosphatase inhibitor. Nucleus extracts were prepared using a KeyGEN nucleus protein extraction kit and protein concentration was quantified using a BCA Protein Assay kit (Beyotime), according to the manufacturer’s instructions. Proteins (30–100 μg) were separated by 12% sodium dodecyl sulfate-polyacrylamide gels electrophoresis (SDS-PAGE) and electrotransferred to PVDF membranes (Millipore Corporation, St. Charles, MO, United States) by a wet transferor (BIO-RAD, United States). Then, PVDF membranes were blocked at room temperature for 2 h in 5% non-fat dry milk in 0.1% Tween-20–Tris buffered saline, pH 7.4 (TBST). After blocking, membranes were incubated overnight with specific primary antibodies at 4°C. Thereafter, membranes were probed with HRP-conjugated secondary antibody for 1 h at room temperature and fluorescence detected with an enhanced chemiluminescence system (ECL; Beyotime). Results were normalized to GAPDH, and band density was analyzed with Image J (National Institutes of Mental Health, Bethesda, MD, United States). Each group was assayed six times (three independent experiments, with two replicates per group).

### Statistical Analyses

One-way ANOVA was used to determine effects of SeMet and/or *E. coli* on LDH activities, cell viability, and protein expression levels in bMECs and macrophages. For all analyses, a least-significant difference test was used to locate differences. All statistical analyses were done with SPSS 23.0 software (SPSS Inc., Chicago, IL, United States) and histograms were produced with GraphPad Prism 7.0 (GraphPad Software, Inc., San Diego, CA, United States). Data were expressed as mean ± standard deviation (SD), with *p* < 0.05, and *p* < 0.01 considered significant and highly significant, respectively.

## Results

### Cytotoxic Effects of Various Concentrations of SeMet on bMEC and Macrophages

**FIGURE 1:**
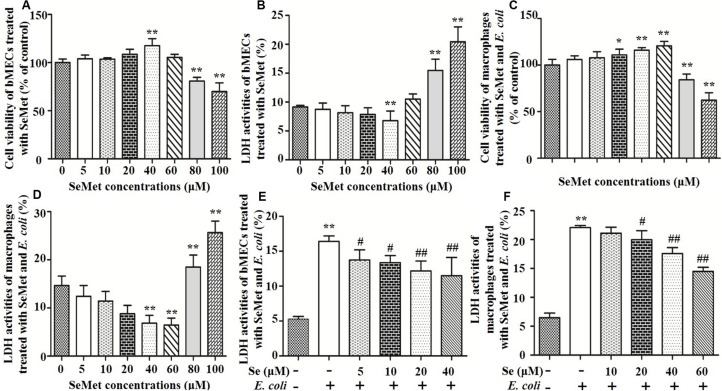
Effects of SeMet and/or ESBL *E. coli* on bMECs and macrophages. bMECs and macrophages were treated with various concentrations of SeMet for 12 h, followed by ESBL-*E. coli* for 4/6 h, respectively. **(A)** Cell viability was determined with an MTT assay in SeMet-treated bMECs. **(B)** LDH release was detected by LDH Assay Kit in SeMet-treated bMECs. **(C)** Cell viability was determined with an MTT assay in SeMet-treated macrophages. **(D)** LDH release was detected with an LDH Assay Kit in SeMet-treated macrophages. **(E)** LDH activities were determined with an LDH Assay Kit in SeMet and ESBL *E. coli*-treated bMECs. **(F)** LDH release was detected with an LDH Assay Kit in SeMet and ESBL *E. coli*-treated macrophages. Data represent means ± SD of five independent experiments. In each independent experiment, there were 10 replicates per group and each sample was assayed in duplicate with similar results. “–” after Se indicated that SeMet was not added. “–” and “+” after *E. coli* indicated that *E. coli* (MOI = 5) were not or were added, respectively. *p < 0.05 and **p < 0.01 were differences compared to the control group; ^#^*p* < 0.05 and ^##^*p* < 0.01 were differences compared to ESBL *E. coli*-infected samples.

To assess cytotoxic effects of selenium (Figure 1), bMECs and macrophages were cultured for 12 h with SeMet at various concentrations and cell viability and LDH activity were measured. Addition of 40 μM SeMet increased viability of bMECs (*p* < 0.01; Figure 1A) and suppressed LDH activities (*p* < 0.01; Figure 1B) compared to the blank control group. In addition, 40 and 60 μM SeMet increased macrophage viability (Figure 1C) and decreased LDH release (Figure 1D) without cytotoxicity (*p* < 0.01) compared to the blank control group. In contrast, 80 and 100 μM SeMet inhibited cell viability and increased LDH activities in both bMECs (*p* < 0.01; Figure 1A) and macrophages (*p* < 0.01; Figure 1B).

### SeMet Reduced LDH Activity After ESBL-*E. coli* Exposure in bMECs and Macrophages

Exposure to ESBL-*E. coli* increased LDH release (*p* < 0.01) compared to the blank control group, which was attenuated (*p* < 0.05) by SeMet treatment in bMECs (5–40 μM; Figure 1E) and macrophages (20–60 μM; Figure 1F) compared to ESBL *E. coli*-infected samples.

### SeMet Increased SeS and Inhibited Protein Expression Through TLR4-Mediated NF-κB Signaling Pathway Induced by ESBL-*E. coli* in bMECs and Macrophages

**FIGURE 2:**
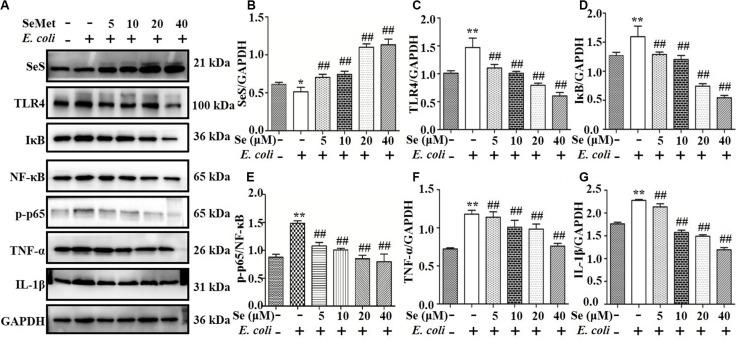
SeMet inhibited activation of TLR4/NF-κB pathway induced by ESBL-*E. coli* in bMECs. bMECs were pre-treated with various concentrations of SeMet for 12 h, followed by ESBL-*E. coli* for 4 h. **(A–G)** Protein levels of SeS, TLR4, IKB, NF-κB, p-p65 (Ser536), TNF-α, and IL-1β were determined by Western blotting. GAPDH was used as loading controls. Data represent means ± SD of three independent experiments. In each independent experiment, there were 10 replicates per group and each sample was assayed in duplicate with similar results. “–” after Se and SeMet indicated that SeMet was not added. “–” and “+” after *E. coli* indicated that *E. coli* (MOI = 5) were not or were added, respectively. *p < 0.05 and **p < 0.01, differences compared to control group; ^##^*p* < 0.01 differences compared to ESBL *E. coli*-infected samples.

**FIGURE 3:**
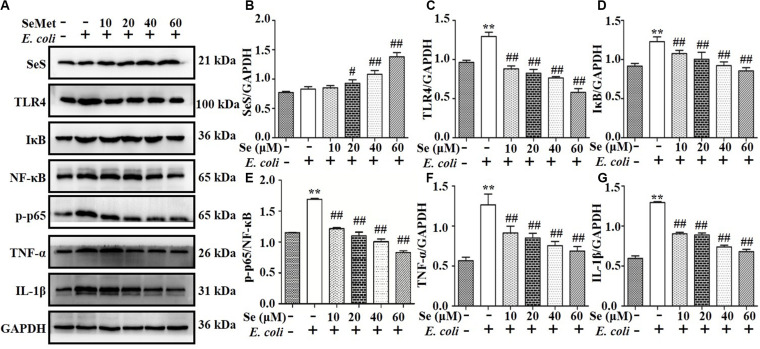
SeMet reversed activation of TLR4/NF-κB pathway induced by ESBL-*E. coli* in macrophages. Macrophages were pre-treated with various concentrations of SeMet for 12 h, followed by ESBL-*E. coli* for 6 h. **(A–G)** Protein levels of SeS, TLR4, IκB, NF-κB, p-p65 (Ser536), TNF-α, and IL-1β were determined by Western blotting. GAPDH was used as loading controls. Data represent means ± SD of three independent experiments. In each independent experiment, there were two replicates per group with similar results. “–” after Se and SeMet indicated that SeMet was not added. “–” and “+” after *E. coli* indicated that *E. coli* (MOI = 5) were not or were added, respectively. **p < 0.01, differences compared to the control group; ^#^*p* < 0.05 and ^##^*p* < 0.01 differences compared to ESBL *E. coli*-infected samples.

Western blotting analysis demonstrated that SeMet increased (*p* < 0.05) ESBL *E. coli*-induced downregulation of SeS in bMECs (5–40 μM; Figures 2A,B) and macrophages (20–60 μM; Figures 3A,B) compared to ESBL *E. coli*-infected samples. Regarding the TLR4-mediated NF-κB signaling pathway, incubation with ST410(4) for 4 or 6 h increased release of IL-1β and TNF-α (Supplementary Figure 1; *p* < 0.01), protein expression of TLR4 (Figures 2A,C, 3A,C), IκB (Figures 2A,D, 3A,D), p-p65 (Figures 2A,E, 3A,E; *p* < 0.01), TNF-α (Figures 2A,F, 3A,F,and Supplementary Figures 1B,D), and IL-1β (Figures 2A,G, 3A,G, and Supplementary Figures 1A,C; *p* < 0.01) and increased nuclear content of NF-κB (Figures 2A,E, 3A,E; *p* < 0.01) compared to the blank control group, whereas pre-treatment with SeMet antagonized this increase in bMECs (5–40 μM; Figure 2 and Supplementary Figure 2A) and macrophages (10–60 μM; Figure 3 and Supplementary Figure 2B) compared to ESBL *E. coli*-infected samples.

### ESBL-*E. coli* Infection Promoted MCP-1, CCL-3, and CCL-5 mRNA Expressions in bMECs

**FIGURE 4:**
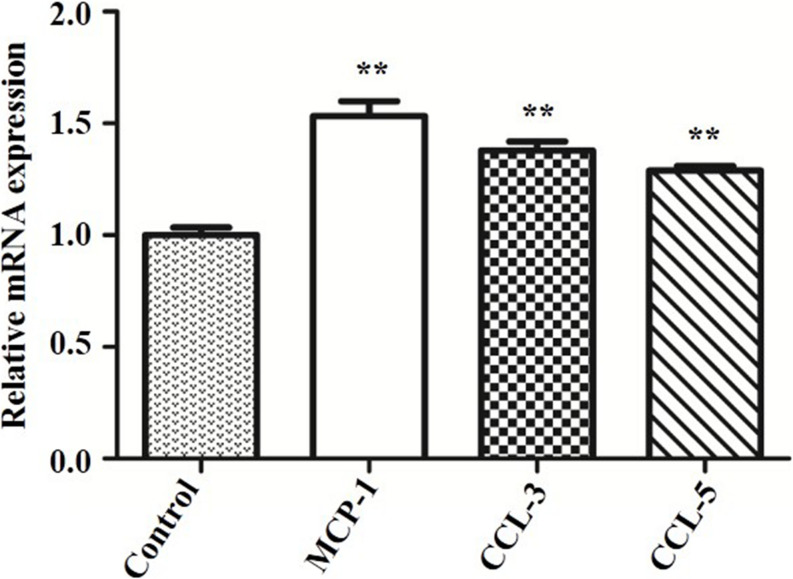
Effect of ESBL *E. coli* on MCP-1, CCL-3, and CCL-5 mRNA expressions in bMECs. bMECs were infected with ESBL *E. coli* (MOI = 5) for 4 h. Data represent means ± SD of three independent experiments. In each independent experiment, there were three replicates per group and each sample was assayed in sextuplicate with similar results. **p < 0.01, differences compared to control group.

To explore why SeMet promoted protein expressions of TLR4-mediated NF-κB signaling pathway in bMECs and macrophages for 4 and 6 h, respectively, MCP-1, CCL-3, and CCL-5 mRNA expressions were analyzed by qRT-PCR. The ESBL-producing ST410(4) *E. coli* increased MCP-1, CCL-3, and CCL-5 mRNA expressions compared to the blank control group (*p* < 0.01) in bMECs (Figure 4).

### SeS Depletion Reversed Effects of SeMet on TLR4-Mediated NF-κB Signaling Pathway in bMECs and Macrophages After ESBL-*E. coli* Infection

**FIGURE 5:**
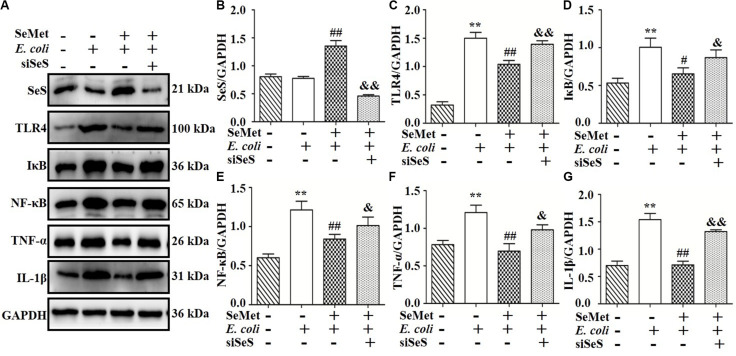
Depletion of SeS reversed effects of SeMet on TLR4/NF-κB pathway in ESBL *E. coli*-treated bMECs. Cells were transfected with control siRNA or SeS siRNA. Subsequently, transfected cells were treated with SeMet for 12 h, followed by ESBL-*E. coli* for 4 h. **(A–G)** Protein levels of SeS, TLR4, IκB, NF-κB, TNF-α, and IL-1β were determined by Western blotting. GAPDH was used as loading controls. Data represent means ± SD of three independent experiments. In each independent experiment, there were two replicates per group with similar results. “–” and “+” after SeMet indicated that SeMet (20 μM) were not or were added, respectively. “–” and “+” after *E. coli* indicated that *E. coli* (MOI = 5) were not or were added. “–” and “+” after siSeS indicated that siSeS (25 nM) were not or were added. **p < 0.01, difference compared to control siRNA transfected samples; ^#^*p* < 0.05 and ^##^*p* < 0.01 differences compared to ESBL *E. coli*-infected control siRNA transfected samples, ^&^*p* < 0.05 and ^&&^*p* < 0.01 differences compared to SeMet and ESBL *E. coli*-treated control siRNA transfected samples.

**FIGURE 6:**
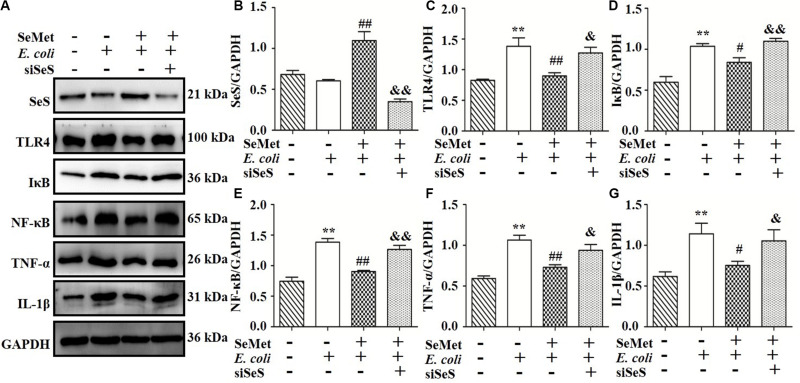
Knockdown of SeS suppressed the modulation of SeMet on TLR4/NF-κB pathway in macrophages infected with ESBL-*E. coli*. Macrophages were transfected with control siRNA or SeS siRNA. Subsequently, transfected cells were treated with CGA for 12 h, followed by ESBL-*E. coli* for 6 h. **(A–G)** Protein levels of SeS, TLR4, IκB, nuclear NF-κB, TNF-α, and IL-1β were determined by Western blotting. GAPDH was used as loading control. Data represent means ± SD of three independent experiments. In each independent experiment, there were two replicates per group with similar results. “–” and “+” after SeMet indicated that SeMet (40 μM) were not or were added, respectively. “–” and “+” after *E. coli* indicated that *E. coli* (MOI = 5) were not or were added. “–” and “+” after siSeS indicated that siSeS (50 nM) were not or were added. **p < 0.01, difference compared to control siRNA transfected samples; ^#^*p* < 0.05 and ^##^*p* < 0.01 differences compared to ESBL *E. coli*-infected control siRNA transfected samples; ^&^*p* < 0.05 and ^&&^*p* < 0.01 differences compared to SeMet and ESBL *E. coli*-treated control siRNA transfected samples.

To clarify whether decreased activation of TLR4-mediated NF-κB signaling pathway induced by SeMet depended on SeS (Figures 5A,B, 6A,B), siRNA of SeS was introduced to bMECs and macrophages induced by ST410(4) and SeMet. Western blotting demonstrated that knockdown of SeS promoted TLR4 (Figures 5A,C, 6A,C), IKB (Figures 5A,D, 6A,D), TNF-α (Figures 5A,F, 6A,F), and IL-1β (Figures 5A,G, 6A,G) protein expressions, as well as nuclear expression of NF-κB (Figures 5A,E, 6A,E) inhibited by SeMet in bMECs (Figure 5) and macrophages (Figure 6) induced by ST410(4) stimulation (4 h).

### NF-κB Involved in SeMet-Regulated Inflammation Induced by ESBL-*E. coli* in bMECs and Macrophages

**FIGURE 7:**
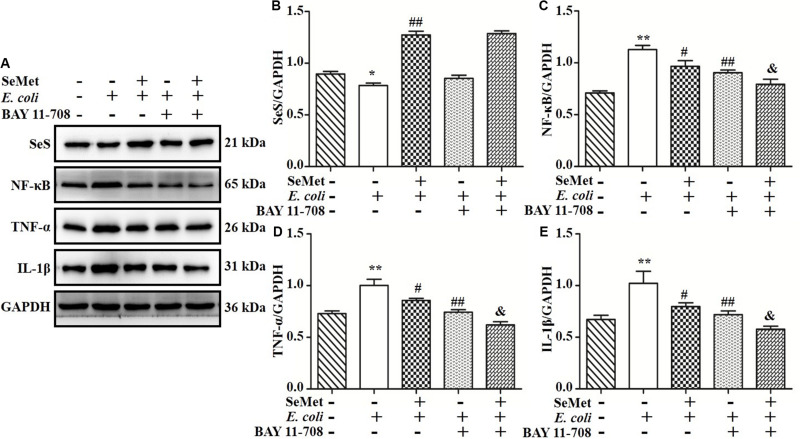
NF-κB mediated regulation of SeMet on inflammation induced by ESBL-*E. coli* in bMECs. The bMECs were incubated with BAY11-708 (10 μM) for 1 h. Subsequently, treated cells were exposed with SeMet for 12 h, followed by ESBL-*E. coli* for 4 h. **(A–E)** Protein levels of SeS, TNF-α, and IL-1β were determined by Western blotting. GAPDH was used as loading control. Data represent means ± SD of three independent experiments. In each independent experiment, there were two replicates per group with similar results. “–” and “+” after SeMet indicated that SeMet (20 μM) were not or were added, respectively. “–” and “+” after *E. coli* indicated that *E. coli* (MOI = 5) were not or were added. “–” and “+” after BAY 11-708 indicated that BAY 11-708 (10 μM) were not or were added. *p < 0.05 and **p < 0.01, differences compared to the control group; ^#^*p* < 0.05 and ^##^*p* < 0.01 differences compared to ESBL *E. coli*-infected samples; ^&^*p* < 0.05 differences compared to SeMet and ESBL *E. coli*-treated samples.

**FIGURE 8:**
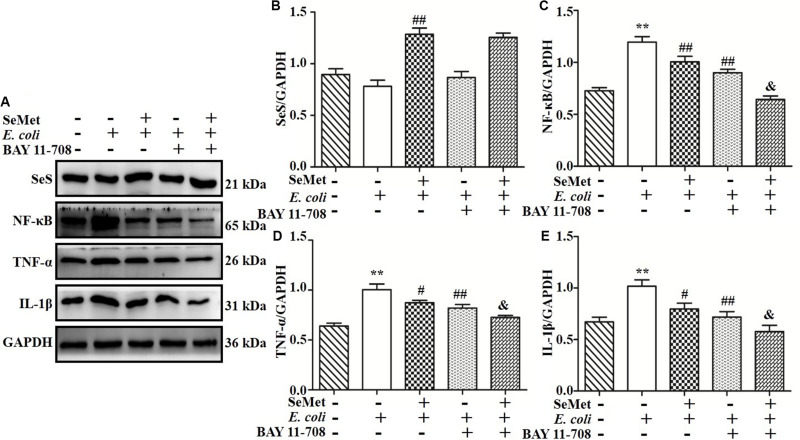
NF-κB mediated decrease of SeMet-induced inflammation in macrophages infected by ESBL-*E. coli*. Macrophages were incubated with BAY11-708 (20 μM) for 1 h. Subsequently, treated cells were exposed with SeMet for 12 h, followed by ESBL-*E. coli* for 4 h. **(A–E)** Protein levels of SeS, TNF-α, and IL-1β were determined by Western blotting. GAPDH was used as loading control. Data represent means ± SD of three independent experiments. In each independent experiment, there were two replicates per group with similar results. “–” and “+” after SeMet indicated that SeMet (40 μM) were not or were added, respectively. “–” and “+” after *E. coli* indicated that *E. coli* (MOI = 5) were not or were added. “–” and “+” after BAY 11-708 indicated that BAY 11-708 (20 μM) were not or were added. **p < 0.01, differences compared to control group; ^#^*p* < 0.05 and ^##^*p* < 0.01 differences compared to ESBL *E. coli*-infected samples; and ^&^*p* < 0.05 difference compared to SeMet and ESBL *E. coli*-treated samples.

To further investigate whether NF-κB pathway is involved in regulation of SeMet on inflammation in ST410(4)-treated bMECs and macrophages, BAY 11-708 (a specific NF-κB inhibitor) was used to suppress NF-κB activation (Figures 7A,C, 8A,C). BAY 11-708 inhibited (*p* < 0.05) TNF-α (Figures 7A,D, 8A,D) and IL-1β (Figures 7A,E, 8A,E) protein levels in bMECs (Figure 7) and macrophages (Figure 8) after ST410(4) treatment. In addition, decreased NF-κB had no effect on SeS protein expression in bMECs or macrophages.

## Discussion

Our aim was to characterize how SeMet protected bMECs and macrophages against inflammation induced by ESBL-*E. coli* infection through modulation of the TLR4/NF-κB signaling pathway. The ESBL-*E. coli* strain ST410(4) isolated from a cow with mastitis enhanced LDH release and protein expression of TLR4, IκB, NF-κB, TNF-α, and IL-1β, whereas SeMet attenuated inflammation in ESBL *E. coli*-treated bMECs and macrophages. In addition, siSeS and BAY 11-708, a NF-κB inhibitor, suppressed NF-κB, TNF-α, and IL-1β expression.

In this study, 40, and/or 60 μM SeMet significantly increased cell viability and inhibited LDH release in bMECs and macrophages, respectively. In contrast, 80 and 100 μM SeMet significantly damaged bMECs and macrophages at 12 h. Therefore, we inferred that although SeMet had protective effects on bMECs (up to 40 μM), and macrophages (up to 60 μM), higher concentrations (i.e., 80 and 100 μM SeMet) had toxic effects on bMECs and macrophages, consistent with another report (Kiełczykowska et al., 2018). In addition, Bi et al. (2016) reported that 4 to 128 μM sodium selenite decreased cell viability in RAW264.7 macrophages at 12 h. Pretreatment of bMECs with 10 μM selenite methionine or 1 μM sodium selenite best rescued cell viability in heat shock-treated bMECs (83.1 and 81.4%, respectively; Zou et al., 2019). This discrepancy might be due to absorptive mechanisms, bioavailability, and toxicity of various Se. In that regard, organic Se has higher bioavailability and less toxicity to animals than inorganic Se (Kim and Mahan, 2001; Weiss and Hogan, 2005). In the current study, SeMet supplementation in advance decreased LDH activities induced by ESBL-producing *E. coli* in bMECs and macrophages. LDH, as a glycolytic enzyme, is abundant and present in most types of mammalian cells; therefore, increased LDH release indicates that cell membranes were damaged (Jin et al., 2013). Thus, we inferred that ESBL-*E. coli* damaged cell membranes, whereas SeMet prevented membrane damage in bMECs and macrophages.

Infection of bMECs and macrophages with *E. coli* incited a rapid inflammatory response, characterized by release of a large number of pro-inflammatory cytokines. Such cytokines activate immune effector cells to eliminate invading pathogen during the first stage (Porcherie et al., 2012; Aribi et al., 2015). Significant increases in TNF-α and IL-1β, important pro-inflammatory cytokines, were induced by *E. coli* in the current study, indicating a profound inflammatory response in bMECs and macrophages. However, expressions of pro-inflammatory cytokines must be tightly regulated and compartmentalized to prevent overexpression of these molecules, with potential for chronic inflammation and tissue injury (Scheibel et al., 2010; Awada et al., 2014). In this study, SeMet inhibited *E. coli*-induced protein expression of TNF-α and IL-1β, indicating that SeMet suppressed inflammation induced by *E. coli* infection in bMECs and macrophages and inhibited the cytokine storm.

Activation of signaling pathways of inflammation has an important role in regulating immune responses (Bi et al., 2016). For example, *E. coli* activated the TLR4/NF-κB signaling pathway in the udder (Yang et al., 2008). Based on significantly increased TLR4, IκB, TNF-α, IL-1β protein expressions, and NF-κB nuclear protein expression induced by *E. coli*, we inferred that it activated TLR4/NF-κB signaling pathways in bMECs and macrophages, consistent with *E. coli* causing mastitis through the TLR4/NF-κB pathway (Ma et al., 2019). Various concentrations of SeMet treatment enhanced SeS protein expression, whereas decreased TLR4, IκB, TNF-α, IL-1β protein expressions, and NF-κB nuclear protein expression in bMECs and macrophages were consistent with a selenium supplement inhibiting LPS-induced inflammatory cytokines through the NF-κB signaling pathway in murine mammary epithelial cells (Zhang et al., 2014). In addition, Se deficiency facilitates inflammation following *Staphylococcus aureus* infection by regulating TLR2/NF-κB pathway in the murine mammary gland and macrophages (Gao et al., 2016; Xu et al., 2020). Thus, we inferred that SeMet promoted SeS expression and relieved *E. coli*-inflammatory responses through TLR4-mediated NF-κB signaling pathway in bMECs and macrophages in this study.

Selenoprotein S, a member of the selenoprotein family, is expressed in multiple tissues and is involved in cellular stress responses and immune and inflammatory processes (Santos et al., 2019). In this study, knockdown of SeS by siRNA partially reversed *E. coli*-induced increases in TLR4, IKB, NF-κB, TNF-α, and IL-1β. Therefore, we concluded that knockdown of SeS inhibited TNF-α and IL-1β expression by mediation of TLR4/NF-κB signaling pathway. Interestingly, the present study clarified that SeMet did not suppress TLR4, IκB, NF-κB, TNF-α, and IL-1β expressions in bMECs and macrophages with SeS depletion. Thus, SeS has an important role in SeMet mediation of the TLR4/NF-κB pathway and in *E. coli*-induced inflammation in bMECs and macrophages. Furthermore, BAY11-708, a NF-κB inhibitor, did not affect SeS expression, and suppressed TNF-α and IL-1β expression in the presence of *E. coli* and/or SeMet, indicating that both SeS and inhibited NF-κB reduced *E. coli*-induced inflammatory response. In addition, although SeS is a target gene of NF-κB (Gao et al., 2006), additional supplemental Se further enhanced SeS expression and the increased SeS had a negative feedback effect on NF-κB activation. Thus, SeMet promoted SeS expression and inhibited *E. coli*-induced inflammation through the TLR4/NF-κB signaling pathway.

Interestingly, SeMet significantly promoted protein expressions of TLR4-mediated NF-κB signaling pathway in bMECs for 4 h, whereas those protein expressions significantly increased in macrophages for 6 h. Perhaps this discrepancy was due to *E. coli* first invading bMECs and subsequently promoting expression of chemotactic factors. Furthermore, those chemotactic factors recruited, and activated macrophages (Swamydas et al., 2015) that phagocytose pathogenic bacteria (e.g., *E. coli*) and mount a partial inflammatory response (Feng et al., 2008). In addition, under physiological conditions, NF-κB is present in the cytoplasm as an inactive heterotrimer consisting of p50, p65, and IκB. However, when activated by various carcinogens, tumor promoters, or proinflammatory agents, ubiquitination and degradation of IκBα promoted NF-κB (p65 subunit) release from the cytoplasm to the nucleus (Alok and Bharat, 2002). However, *E. coli* significantly increased IκB and NF-κB protein expressions in this study. This apparent discrepancy may have been due to increased nuclear NF-κB driving IκB expression, generating a negative feedback loop (Hayden and Ghosh, 2008). Moreover, SeMet treatment significantly decreased IκB and NF-κB protein expressions in this study, indicating that SeMet may have enhanced the negative feedback effect of NF-κB activation on IκB expression. However, the mechanism by which SeMet inhibited NF-κB expression with decreased IκB expression remains unclear and warrants further study.

## Conclusion

**FIGURE 9:**
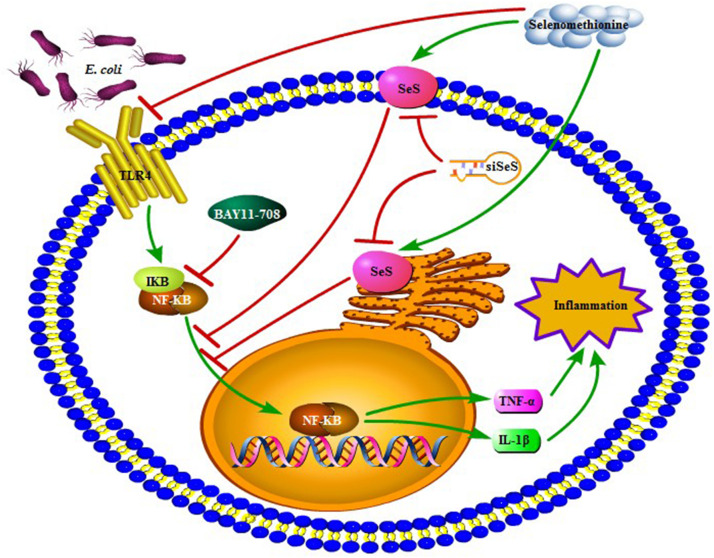
A proposed signaling pathway involved in SeMet against ESBL *E. coli*-induced inflammation. Note that SeMet protects bMECs and macrophages against inflammation by inhibition of TLR4/NF-κB signaling pathway. Meanwhile, SeMet suppressed NF-κB pathway through SeS and inhibited NF-κB-reduced inflammation. Green arrow and red bar indicate stimulation and inhibition, respectively.

Extended-spectrum β-lactamase *E. coli* induced expressions of chemotactic factors in bMECs and then recruited and activated macrophages. In addition, SeMet attenuated ESBL *E. coli*-induced inflammation through activated SeS-mediated TLR4/NF-κB signaling pathway in bMECs and macrophages (Figure 9), which provided experimental evidence for cytoprotective roles of Se.

## Data Availability Statement

The raw data supporting the conclusions of this article will be made available by the authors, without undue reservation, to any qualified researcher.

## Author Contributions

BH conceived and designed the experiments. CZ, GL, MZ, SX, and JG performed the experiments, analyzed the data, and contributed to drafting the manuscript. SR, YL, and GL analyzed the data, participated in the preparation of the manuscript, and analyzed the results. CZ, HB, and JK wrote, reviewed, and edited the manuscript. All authors approved the final version of the manuscript.

## Conflict of Interest

The authors declare that the research was conducted in the absence of any commercial or financial relationships that could be construed as a potential conflict of interest.
