# Supplementary material for: Selenomethionine Suppressed TLR4/NF-κB Pathway by Activating Selenoprotein S to Alleviate ESBL Escherichia coli-Induced Inflammation in Bovine Mammary Epithelial Cells and Macrophages
Source: Front Microbiol. 2020 Jul 8;11:1461. doi: 10.3389/fmicb.2020.01461 (PMC7360804; doi:10.3389/fmicb.2020.01461)
Supplement: Supplementary file 1 [file Table_1.DOC]

**Supplemental Materials**


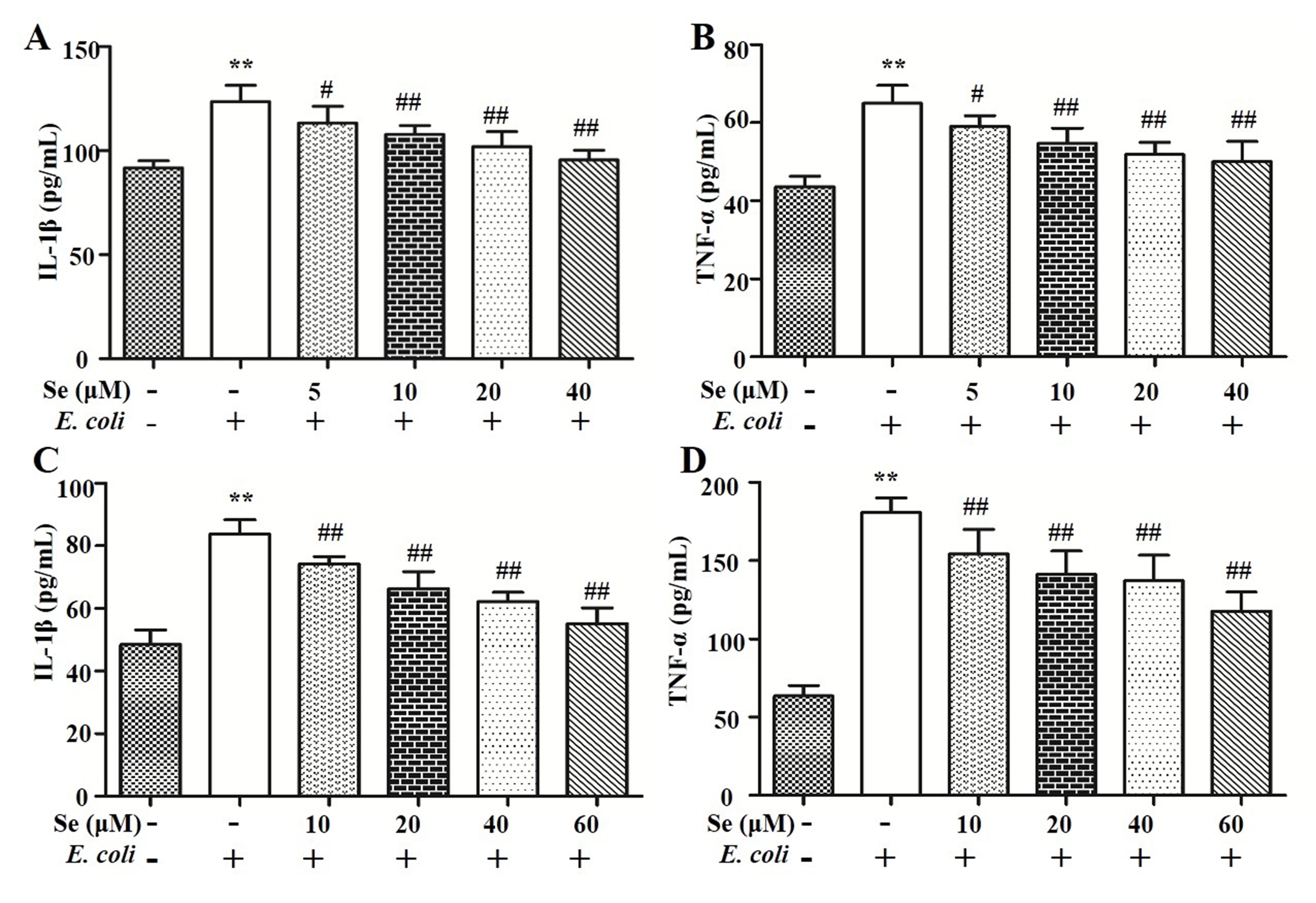


**SUPPL FIGURE 1׀ SeMet inhibited the increase of IL-1β and TNF-α induced by ESBL-*E. coli* in bMECs (A and B) and macrophages (C and D).** bMECs and macrophages were treated with various concentrations of SeMet for 12 h, followed by ESBL-*E. coli* for 4/6 h, respectively. Data represent the means ± SD of 5 independent experiments. In each independent experiment, there were 10 replicates per group and each sample was assayed in duplicate with similar results. “–” after Se indicated that SeMet was not added. “–” and “+” after *E. coli* indicated that *E. coli* (MOI=5) were not or were added, respectively. ** *p* < 0.01 indicated significant differences compared to control group; # *p* < 0.05 and ## *p* < 0.01 differences compared to ESBL *E. coli*-infected samples.


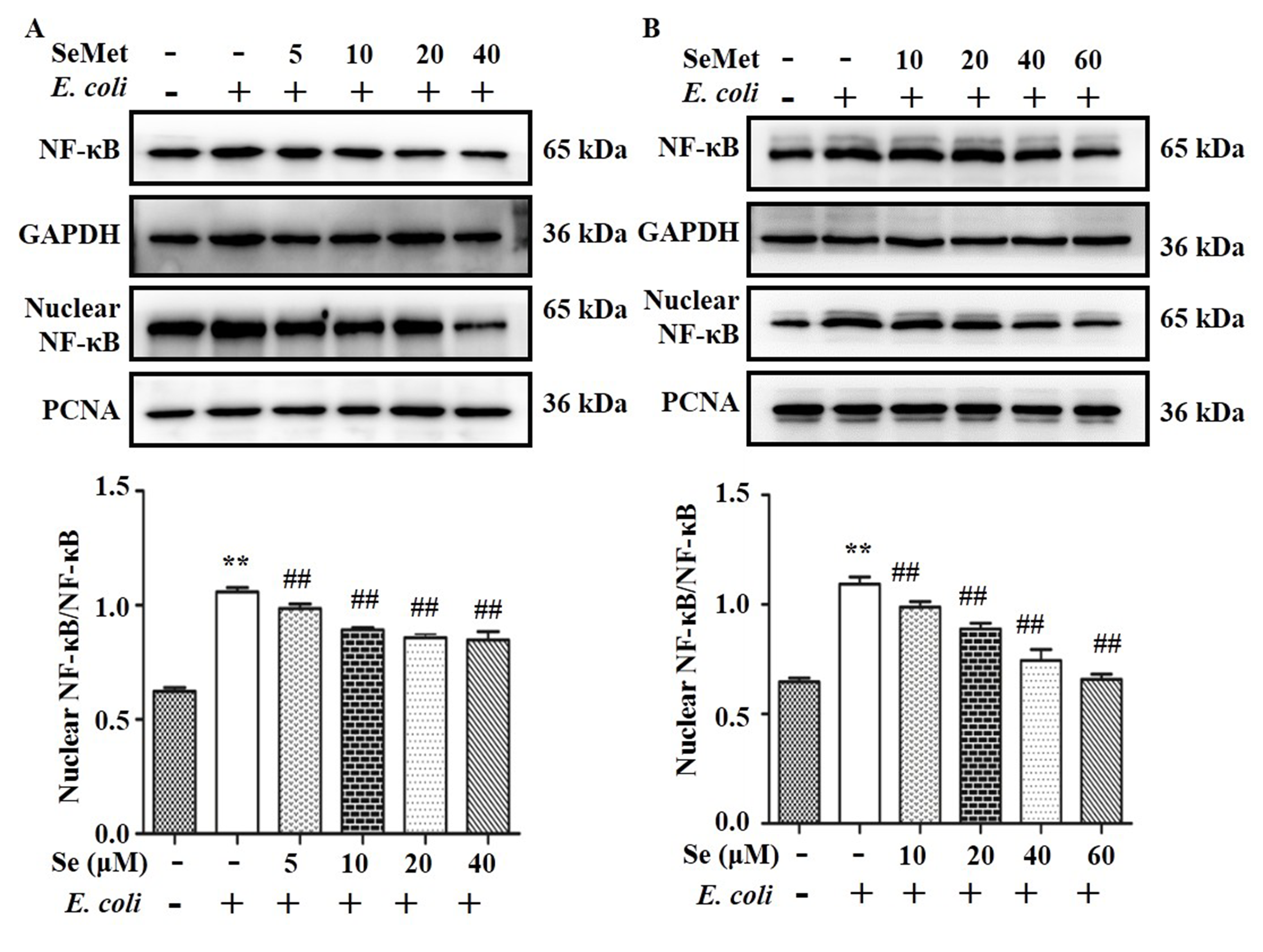


**SUPPL FIGURE 2׀ SeMet inhibited nuclear protein expression of NF-****κB induced by ESBL-*E. coli* in bMECs (A) and macrophages (B).** bMECs and macrophages were pre-treated with various concentrations of SeMet for 12 h, followed by ESBL-*E. coli* for 4/6 h. GAPDH and PCNA were used as loading controls. Data represent means ± SD of 3 independent experiments. In each independent experiment, there were 10 replicates per group and each sample was assayed in duplicate with similar results. “–” after Se and SeMet indicated that SeMet was not added. “–” and “+” after *E. coli* indicated that *E. coli* (MOI=5) were not or were added, respectively. ** *p* < 0.01, indicated differences compared to control group; ## *p* < 0.01 indicated differences compared to ESBL *E. coli*-infected samples.
